# Smoking as an Independent Risk Factor for Hepatocellular Carcinoma Due to the α7-Nachr Modulating the JAK2/STAT3 Signaling Axis

**DOI:** 10.3390/jcm8091391

**Published:** 2019-09-05

**Authors:** Ching-Li Li, Yen-Kuang Lin, Hsin-An Chen, Chien-Yu Huang, Ming-Te Huang, Yu-Jia Chang

**Affiliations:** 1 Graduate Institute of Clinical Medicine, College of Medicine, Taipei Medical University, Taipei 110, Taiwan; d118103007@tmu.edu.tw; 2 Department of Surgery, Sijhih Cathay General Hospital, New Taipei City 221, Taiwan; 3 Biostatistics Center, Taipei Medical University, Taipei 110, Taiwan; robbinlin@tmu.edu.tw; 4 Department of Surgery, Taipei Medical University, Shuang Ho Hospital, New Taipei City 235, Taiwan; speedupplus@gmail.com (H.-A.C.); cyh@tmu.edu.tw (C.-Y.H.); 5 Division of General Surgery, Department of Surgery, School of Medicine, College of Medicine, Taipei Medical University, Taipei 110, Taiwan; 6 International PhD Program in Medicine, Taipei Medical University, Taipei 110, Taiwan; 7 Cell Physiology and Molecular Image Research Center, Wan Fang Hospital, Taipei Medical University, Taipei 116, Taiwan; 8 Cancer Research Center and Translational Laboratory, Department of Medical Research, Taipei Medical University Hospital, Taipei Medical University, Taipei 110, Taiwan

**Keywords:** hepatocellular carcinoma (HCC), cigarette smoking, nicotine, α7-nicotinic acetylcholine receptor (α7-nAChR), metastasis, recurrence, TGR5, JAK2-STAT3 signaling, CSC

## Abstract

Background: Hepatocellular carcinoma (HCC) is a worldwide health problem. Currently, there is no effective clinical therapeutic strategy for HCC. Smoking is associated with several malignant diseases including cancers. Experimental approach: However, the impact of smoking on HCC is still unresolved. Retrospectively reviewed HCC patients diagnosed between 1 January 2010 and 31 December 2015 at Taipei Medical University-Shuang Ho Hospital (Ministry of Health and Welfare). We found that smoking was associated with a poor prognosis, especially recurrence and patient survival after curative surgery using a clinicopathological analysis. Results: Our univariate and multivariate analyses showed that the α7-nicotinic acetylcholine receptor (α7-nAChR) was an oncogene and risk factor for post-resection recurrence. The α7-nAChR was overexpressed in HCC tissues compared to their non-tumor counterparts. Silencing the α7-nAChR reduced the viability of HCC cells, suppressed cellular proliferation, attenuated migration and invasion, and diminished the tumor’s sphere-formation ability, with concurrent downregulation of expression levels of the TGR5, p-JAK2, p-STAT3 (Tyr705/Ser727), RhoA, ROCK1, MMP2, and MMP9 proteins. Furthermore, a positive correlation was found between α7-nAChR and JAK2 expressions (*p* = 0.01) in HCC specimens, as well as their membranous co-localization. Conclusion: Together, we demonstrated that the α7-nAChR may be an independent prognosticator of the progression and prognosis of HCC patients. These findings suggest that the α7-nAChR drives the progression and recurrence of HCC through JAK2/STAT3 signaling and is a novel target for anti-HCC therapy.

## 1. Introduction

Hepatocellular carcinoma (HCC) is one of the most-often diagnosed malignancies and ranks as the second leading cause of cancer-related deaths worldwide [1]. Many risk factors of HCC attributable to high- and low-risk populations also differ [2]. In high-risk populations of China and Africa, chronic infection with the hepatitis B virus (HBV) is the most important risk factor [3]. Conversely, in low-risk counties in the west such as the United States, chronic alcohol abuse is the most important contributing factor [4].

Despite advances in earlier screening methods, and improved diagnostic techniques and treatment modalities, the prognosis of patients with HCC remains poor according to high recurrence rates of 50–60% at 3 years and 70–100% at 5 years [5], and extrahepatic metastasis (EHM) rates of 15–17% [6,7], even after curative liver resection. Currently, the optimal treatment of recurrent HCC and the molecular mechanisms underlying the occurrence of HCC relapse remain unclear, thus necessitating the discovery and/or development of relatively cheaper and more-efficacious therapeutic strategies.

The association between cigarette smoking and HCC development is independent of geography and racial ethnicity. Smoking causes a variety of adverse effects on the liver such as liver carcinogens, as has been reported. Smoking is related to the recurrence of gastric, colon, pancreatic, prostate, and bladder cancers [8,9,10]. Recently many studies reported the negative impacts of cigarette smoking and poor prognoses of HCC patients [11]. Despite the cloud of ambiguity, cigarette smoking was recently touted as an independent risk factor for HCC initiation [12]. After surgery, continued smoking was also suggested to be strongly correlated with disease recurrence and compromise of the survival of HCC patients with HBV [13]. Nevertheless, the biological effects and molecular mechanisms of smoking in HCC remain obscure. Conversely, smoking was shown to lack an independent association with HCC survival in a multivariate model and was in fact associated with favorable prognostic features in patients with HCC [14]. The current smoking habit at the time of surgical treatment is a risk factor for poor long-term survival in non-B non-C (NBNC) HCC patients. Current smokers tend to have multiple HCCs at a younger age than other patients [15]. A smoking habit is significantly correlated with the overall and disease-specific survivals of patients with HCV-related HCC [16].

The major addictive bioactive component of cigarettes is nicotine, the bio-effects of which are mediated by nicotinic acetylcholine receptors (nAChRs) [17,18,19,20]. Among the various types of nAChRs, the oncogenic role of the α7-nAChR in several malignancies, including lung cancer [21], malignant pleural mesotheliomas [22], and breast cancer [23], has garnered increased interest. Most interestingly is growing evidence that the α7-nAChR mediates regulation of self-renewal and cellular differentiation, with its presence in mature tissues and organs, and in undifferentiated stem progenitor cells, especially with suggested implication in the proliferation of cancer stem cells (CSCs) in breast cancer [24].

TGR5, a G-protein-coupled bile acid cell surface receptor (GPBAR1), which is ubiquitously distributed in the human body and highly expressed in several tissues including the spleen, placenta, and stomach, is known to be involved in biological processes regulated by bile acids, in health and disease [25]. Bile acid-activated TGR5 induces the production of cyclic adenosine monophosphate (cAMP), and modulates the activities of several signaling pathway effectors, including AKT, NF-kB, and ERK [26], as well as mediates the immunosuppressive activities of bile acids via suppression of lipopolysaccharide (LPS)-induced pro-inflammatory cytokines, including interleukin-1α (IL-1α), IL-1β, IL-6, and tumor necrosis factor-α (TNF-α) in a NF-kB-dependent manner (reviewed in [27]). These activities are cancer-relevant; however, the association of TGR5 with α7-nAChR and its probable role in development, progression, or prognosis of HCC remains unclear. Furthermore, it is increasingly evident that the JAK2/STAT3 signaling pathway plays a critical role in tumor growth, proliferation, and the metastasis of several malignancies, including lung cancer [28]. The JAK2-modulated transcription factor STAT3, involved in immune responses, inflammatory processes, and cancer initiation, is activated by several cytokines, growth factors, and oncogenes. It has been suggested that by suppressing STAT3 signaling, TGR5 may inhibit chemically induced inflammation-related liver cancer [29]. However, in the light of the anti-inflammatory role and oncogenic roles of TGR5 and STAT3, respectively, we thus investigated the hitherto unexplored probable oncogenic role of TGR5 and its modulation of the JAK2/STAT3 signaling in HCC progression and prognosis.

However, the role of the α7-nAChR in HCC remains unclear; in the present study, we investigated the functional relevance of the α7-nAChR in HCC progression and prognosis, as well as unraveled the underlying molecular mechanism for its probable oncogenic role in patients with HCC. In addition, in vitro assays were used to study the functional role of the α7-nAChR. Our results showed that high expression of the α7-nAChR promotes the invasion, recurrence, and poor prognosis in HCC.

## 2. Materials and Methods

### 2.1. Study Design

HCC patients diagnosed between 1 January 2010 and 31 December 2015 at Taipei Medical University-Shuang Ho Hospital (Ministry of Health and Welfare) were retrospectively evaluated. All patients provided informed consent after admission and before surgery. Information on the smoking status and smoking quantity (pack-years, PY) was reviewed. Following the definition of current smokers commonly used in the tobacco field, we defined current smokers as those who had smoked at least 100 cigarettes in their lifetimes and smoked either daily or occasionally at the time of the survey. Former smokers were defined as those who had smoked at least 100 cigarettes but had not smoked for at least 1 year prior to the survey. Non-smokers were defined as those who had smoked fewer than 100 cigarettes in their lifetimes. Factors affecting the recurrence, metastasis, and overall survival (OS) were analyzed, and risk factors were compared. Those significant factors were further analyzed for α7-nAChR expression by immunohistochemical (IHC) staining and its correlation with clinicopathological features. This study was approved by the Institutional Human Research Ethics Review Board (N201602072) of Taipei Medical University.

### 2.2. Patients’ Tissue Specimens

This study retrospectively analyzed tissue samples from an HCC cohort of HCC patients from Taipei Medical University Shuang Ho Hospital (*n* = 179) diagnosed between 1 January 2010 and 31 December 2015. Relevant clinicopathological data were retrospectively obtained from clinical and pathology report archives. Fresh HCC tissue samples and paired adjacent non-cancerous tissues from each patient were collected from HCC curative resection surgery, snap-frozen, and stored at −80 °C until used for experimental purposes. All patients were followed up for ≥36 months.

### 2.3. Reagents

An anti-GPCR TGR5 (ab72608 rabbit polyclonal antibody (pAb)) antibody was purchased from Abcam (Biochiefdom International, New Taipei City, Taiwan). Antibodies against RhoA (ab187027 rabbit monoclonal antibody (mAb)), ROCK1 (ab45171 rabbit mAb), matrix metalloproteinase 2 (MMP2; ab37150 rabbit pAb), and MMP9 (ab38898 rabbit pAb) were also purchased from Abcam, Cambridge, United Kingdom. Anti-phospho-Janus kinase 2 (JAK2; Tyr1007/1008: #3771 rabbit mAb), anti-JAK2 (D2E12: #3230 rabbit mAb), anti-phospho-signal transducer and activator of transcription 3 (STAT3; Tyr705; D3A7: #9145 rabbit mAb), anti-phospho-STAT3 (Ser727; D4X3C: #34911 rabbit mAb), and anti-STAT3 (D3Z2G: #12640 rabbit mAb) were purchased from Cell Signaling Technology (CST, Beverly, MA, USA), and the β-actin (C4: sc-47778) antibody was purchased from Santa Cruz Biotechnology (Santa Cruz, CA, USA). Alexa Fluor 647 donkey anti-rabbit immunoglobulin G (IgG) and Alexa Fluor 488 donkey anti-rabbit IgG were purchased from Invitrogen (Grand Island, NY, USA).

### 2.4. Cell Lines and Cell Culture

The human Hep-J5 and Mahlavu HCC cell lines were established by Dr. C.S. Yang as previously described (Wang et al. [30]) and were cultured in Dulbecco’s modified Eagle’s medium (DMEM, Invitrogen, Life Technologies, Carlsbad, CA, USA), supplemented with 10% fetal bovine serum (FBS) and 1% penicillin–streptomycin (Invitrogen, Life Technologies) at 37 °C, in a 5% humidified CO_2_ incubator. Cells were subcultured at 80–90% confluence.

### 2.5. Small Hairpin (sh)RNA Transfection

α7-nAChR-knockdown (KD) Hep-J5 or Mahlavu cells were established by an shRNA method, as previously described [28,29]. Stably transfected clones were then selected using 10 µg/mL puromycin and used for reverse-transcription polymerase chain reaction (RT-PCR) or Western blot analyses to confirm expression of the α7-nAChR.

### 2.6. Analyses of an Online Cancer Microarray Dataset

The Gene Expression Omnibus (GEO) human liver cancer microarray dataset consisting of 38 HCC samples and 19 normal liver cases was analyzed for expressions of α7-nAChR (CHRNA7) and JAK2 genes as performed on the Oncomine platform (https://www.oncomine.org/resource/).

### 2.7. Sulforhodamine B (SRB) Cell Viability Assay 

Hep-J5 wild-type (WT) or α7-nAChR-KD cells were seeded at a density of 3 × 10^3^ cells/well in 96-well plates, then incubated in humidified 5% CO_2_ at 37 °C for 24 or 48 h. After 24 or 48 h, HCC cells were fixed in 10% trichloroacetic acid (TCA), then washed with double-distilled (dd)H_2_O, before viable cells were stained with 0.4% SRB in 1% acetic acid. The free dye was removed by repeated washings with 1% acetic acid before air-drying the plates, while the bound dye was dissolved in 10 mM Trizma, and the absorbance was read at a 495-nm wavelength in a microplate reader.

### 2.8. Immunoprecipitation and Western Blot Analysis

Cultured HCC cells were harvested, washed with ice-cold phosphate-buffered saline (PBS), and lysed using an ice-cold lysis buffer solution. The cell lysate was then immunoprecipitated with 10 μg/mL anti-α7-nAChR or anti-Jak2 monoclonal antibodies at 4 °C for 2 h and precipitated by following the protocol of the Pierce™ Co-Immunoprecipitation Kit. After boiling for 5 min, the immunoprecipitated protein/protein lysate was immunoblotted, and separated proteins were transferred to polyvinylidene difluoride (PVDF) membranes (Millipore, Burlington, MA, USA). The membranes were blocked with 5% non-fat milk in TBST for 1 h at room temperature before incubation overnight at 4 °C with primary antibodies against anti-α7-nAChR (1:1000), TGR5 (1:1000), RhoA (1:1000), ROCK1 (1:1000), MMP2 (1:1000), MMP9 (1:1000), p-JAK2 (1:1000), JAK2 (1:1000), p-STAT3 (Tyr705) (1:1000), p-STAT3 (Ser727) (1:1000), STAT3 (1:1000), and β-actin (1:500). The membranes were then washed with TBST, incubated with a secondary antibody labeled with horseradish peroxidase (HRP) (1:1000) for 1 h at room temperature, and washed again with TBST. Bands were detected using enhanced chemiluminescence (ECL) Western blotting reagents, and imaging was performed in the BioSpectrum Imaging System (UVP, Upland, CA, USA).

### 2.9. RT-PCR

Total RNA was extracted from Hep-J5 or Mahlavu scrambled control and α7-nAChR-KD cells using Trizol reagent according to the manufacturer’s instructions. The extracted RNA was reverse transcribed using the Quantscript RT kit following the manufacturer’s protocol. The RT-PCR was performed using 50 μL of reaction mix that contained 1 μg of complementary (c)DNA as a template, 1 μM of specific oligonucleotide primers, and 25 μL Taq mixture containing 0.5 units of Taq DNA polymerase. PCR products were separated by electrophoresis in a 2% agarose gel using 1× TAE buffer.

### 2.10. Wound Healing Migration Assay

Cells were seeded in six-well plates (Corning, Corning, NY, USA) with DMEM containing 10% FBS and cultured to 95–100% confluence. A scratch along the median axis was then made with a sterile yellow pipette tip across the cells. Cell migration pictures were captured at 0 and 48 h after the medium scratch, under a microscope and analyzed with NIH Image J software (https://imagej.nih.gov/ij/download.html).

### 2.11. Matrigel Invasion Assay

Cells (2 × 10^5^) were seeded in 24-transwell chambers with an 8-μm pore membrane coated with Matrigel in the upper chamber of the transwell system containing serum-free DMEM. The lower chamber of the transwell chamber contained medium with 20% FBS. After incubation at 37 °C for 6 h, non-invaded HCC cells on the upper side of membrane were carefully removed with a cotton swab, while the invaded cells were stained with crystal violet dye, air-dried, and photographed under a microscope. Images were analyzed with NIH Image J software (https://imagej.nih.gov/ij/download.html).

### 2.12. Sphere Formation Assay

Cells (5 × 10^3^ per well) were plated in ultra-low-attachment six-well plates (Corning) containing stem cell medium consisting of serum-free DMEM supplemented with 10 ng/mL human basic fibroblast growth factor (bFGF; (Invitrogen, Grand Island, NY, USA), 1× B27 supplement, and 20 ng/mL epidermal growth factor (EGF; Invitrogen). The medium was changed every 72 h. After 14 days of incubation, formed spheres were counted and photographs taken.

### 2.13. Immunohistochemistry (IHC) and Immunofluorescence (IFC) Staining

For the IHC assay, HCC tissue sections were incubated with primary antibodies against the α7-nAChR (1:200 dilution) or JAK2 (1:200 dilution) overnight at 4 °C, then incubated with a biotin-labeled secondary antibody (1:100 dilution) for 1 h at room temperature. Tissue sections were then incubated with ABC-peroxidase and diaminobenzidine (DAB), counterstained with hematoxylin, and visualized using light microscopy at 200× magnification. For IFC staining, tissue sections were fixed with 4% paraformaldehyde, washed with PBS three times, and then permeabilized with 0.1% a Triton X-100/PBS solution for 10 min before incubating with primary antibodies against the α7-nAChR or JAK2, followed by incubation with a goat anti-mouse Alexa Fluor488 secondary antibody (Thermo Fisher Scientific, Eugene, OR, USA). Nuclear staining used 4′,6-diamidino-2-phenylindole (DAPI) (Molecular Probes, Thermo Fisher Scientific). Tissue staining visualization and imaging were done under a Nikon E800 fluorescent microscope. Ten representative staining fields of each section were analyzed for α7-nAChR expression by two independent pathologists without knowledge of patient characteristics. α7-nAChR staining was determined by a Q-score. With the quantitative and qualitative scoring systems, the absolute quantity of positively stained cells was counted for each IHC slide and IHC staining intensity. Score “0” indicated negative staining and negative positive staining cells; score “1” indicated weak staining and <10% positive staining cells; score “2” indicated moderate staining and 10–50% positive staining cells; and score “3” indicated strong staining and >50% positive staining cells. The percentage of cells with a positive score in the membrane was determined. Results were scored by multiplying the percentage of positive cells (P) by the intensity (I) using the formula Q = P × I, where the maximum score was 300. We also evaluated percentage scores from 0 to 100%. Finally, we measured the total IHC score by intensity × percentage, then 50% cut-off for high expression and low expression groups.

### 2.14. Statistical Analysis

All assays were performed at least thrice in triplicate. Values were expressed as the mean ± standard deviation (SD). Comparisons between groups were estimated using Student’s *t*-test for cell line experiments or the Mann–Whitney U-test for clinical data, Spearman’s rank correlation between variables, and the Kruskal–Wallis test for comparison of three or more groups. The Kaplan–Meier method was used for the survival analysis, and the difference between survival curves was tested by a log-rank test. Univariate and multivariate analyses were based on the Cox proportional hazards regression model. All statistical analyses were performed using IBM SPSS Statistics for Window, version 20 (IBM, Armonk, NY, USA). A *p* value < 0.05 was considered statistically significant.

## 3. Results

### 3.1. Patient Characteristics

The study cohort consisting of 179 HCC patients (143 males and 36 females) with a median age of 62.5 (range, 36–87) years was divided into non-smokers (*n* = 90), (*n* = 36), and current smokers (*n* = 53) (Table 1). According to the UICC/AJCC staging system, 65 patients (36.3%) were in stage I, 59 (33.0%) were in stage II, 46 (25.7%) were in stage III, and nine (5.0%) were in stage IV. Pathological features included the α-fetoprotein (AFP) status (69 patients (38.5%) had levels of ≥20 ng/mL and 110 (61.5%) had levels of <20 ng/mL), tumor size (64 patients (35.8%) had a size of <5 cm and 115 (64.2%) had a size of ≥5 cm), tumor number (110 (61.5%) had solitary tumors and 69 (38.5%) had multiple tumors), and vascular invasion (107 (59.8%) had vascular invasion and 72 (40.2%) did not). Among the 179 patients, 53 (29.6%) were current smokers, 87 (48.6%) were alcohol consumers, 84 (46.9%) were hepatitis B surface antigen (HbsAg) positive, and 25 (14.0%) were hepatitis C virus (HCV) positive.

### 3.2. α7-nAChR Is Nicotine-Dependent and Deferentially Expressed in HCC and Non-Tumor Liver Tissues

**Figure 1:**
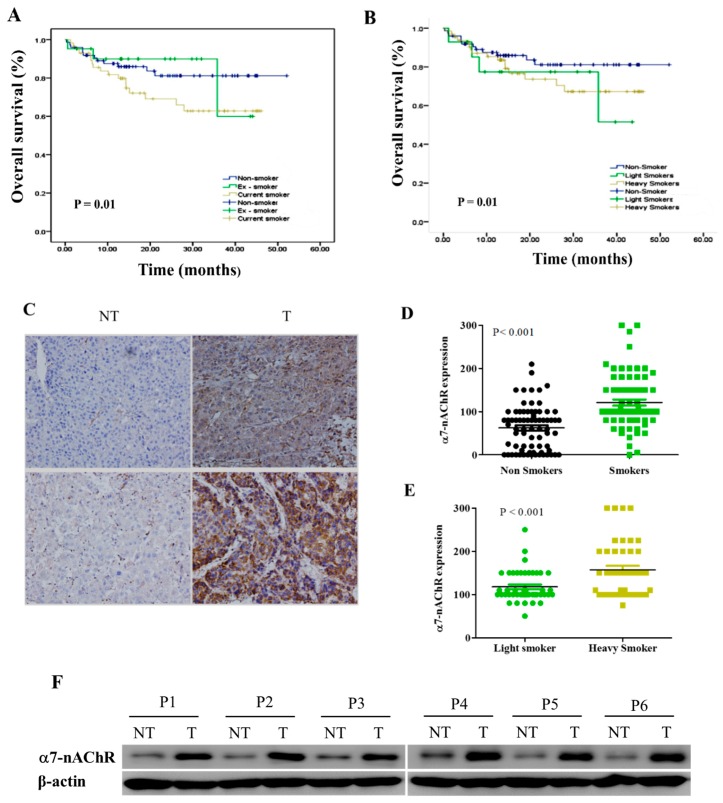
The α7-nicotinic acetylcholine receptor (α7-nAChR) is nicotine-dependent and deferentially expressed between hepatocellular carcinoma (HCC) and non-tumor (NT) liver tissues. Kaplan–Meier plots show effects of (**A**) the smoking status and (**B**) number of cigarettes on the overall survival of patients with HCC. (**C**) IHC staining of the α7-nAChR showed differential expressions of the α7-nAChR in non-tumor liver and HCC tissues. Original magnification × 200 (upper panel). Western blot image showing that α7-nAChR protein expression is upregulated in HCC primary culture cells compared to their non-tumor liver counterparts (lower panel). (**D**) The α7-nAChR is highly expressed in HCC patients who are smokers compared to non-smokers. (**E**) Light smokers expressed less α7-nAChR compared to heavy smokers among patients with HCC. (**F**) Western blot analysis of α7-nAChR protein expression in 6 pairs of HCC tissues (T) and non-tumor tissues (N), using β-actin as an endogenous control.

Based on convergent evidence that smoking is a risk factor in the development of HCC [11,12,13], a preliminary evaluation of the effect of smoking on the survival status of our patients with HCC revealed that at 36 months post-resection, patients with HCC who were non-smokers had 20% and 31% survival advantages, respectively, over those who were current smokers (Figure 1A). Based on the smoking pack-year, light smokers (<20 packs of cigarettes/year) and non-smokers exhibited 78% and 84% OS rates, respectively, compared to 70% in heavy smokers (≥20 packs of cigarettes/year) (Figure 1B). To investigate the role of the α7-nAChR in HCC progression, we examined α7-nAChR expression in 45 paired tumor tissues and adjacent non-cancerous tissues by IHC. Results of our IHC staining showed moderate to strong α7-nAChR expression in HCC tissues compared to the no or only mild expression in non-tumor liver tissues (Figure 1C, upper). By Western blotting of frozen tumor samples and adjacent non-tumorous liver tissues (Figure 1C, lower), α7-nAChR protein expression was significantly upregulated in tumor tissues. The mean IHC score in tumor tissues was 180.7 ± 5.6, which was significantly greater than that (59.8 ± 5.9) of adjacent normal liver tissues (*p* <0.01). We also examined the differential expression of the α7-nAChR in smoking and non-smoking HCC patients. In non-smoker tissues, the mean positive staining was 84 ± 3.9, significantly lower than that of smoker HCC tissues with a mean of 160.4 ± 8.6 (*p* < 0.001, t test) (Figure 1D). In addition, light smokers exhibited a mean staining intensity of 117.8 ± 5.4, compared to 157.2 ± 9.4 for heavy smokers (*p* < 0.001, t test) (Figure 1E). The protein level of α7-nAChR was confirmed in all the six paired tissue samples by western blot analysis. Results revealed that all examined HCC samples showed a higher expression level of α7-nAChR protein, which was consistent with the IHC staining level (Figure 1F). These findings indicated that α7-nAChR was commonly upregulated in either HCC tissues or cell lines.

### 3.3. α7-nAChR Expression Is an Independent Prognosticator in Patients with HCC

**Figure 2:**
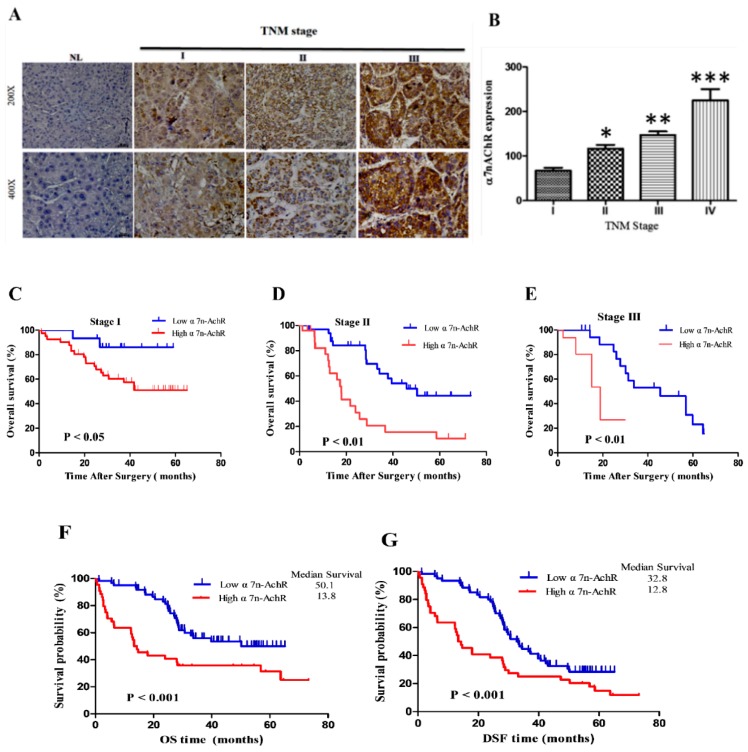
α7-Nicotinic acetylcholine receptor (α7-nAChR) expression is a prognosticator of patients with hepatocellular carcinoma (HCC). (**A**) Representative images of α7-nAChR expression levels in HCC tissues of different TMN stages showing mild expression in stage I and II cases but high expression in stage III, compared to no expression in non-tumor liver (NL) tissues. The original magnification is indicated; bar = 100 μm. (**B**) The IHC Q-score of α7-nAChR expression in samples displayed in A. The mean ± SD was used to express the data. * *p* < 0.05. Graphical representation of the positive correlation of α7-nAChR expression levels and TMN stage. * *p* < 0.05, ** *p* < 0.01, *** *p* < 0.001. (**C**–**E**) Kaplan-Meier survival plots showing the effect of the α7-nAChR on patients with HCC in different TMN stages. (**F**,**G**) Kaplan–Meier survival curves for overall survival (OS) and disease-free survival (DFS) of patients with low versus high expression of the α7-nAChR.

We further evaluated the pathological significance of α7-nAChR expression levels, and an IHC staining analysis was carried out on paraffin-fixed HCC tissues of 179 patients. Immunoreactivity for the α7-nAChR was mainly at the cytomembrane, and dark-brown immunostaining was mostly prevalent in cancer cells. We further evaluated the pathological significance of α7-nAChR expression levels in 179 HCC patients with IHC. According to the scoring system, we divided patients into two groups, with low expression (with scores of 0 and 1) and high expression (with scores of 2 and 3). Of the total 179 patients, 61.5% (110 of 179) showed high α7-nAChR expression (Table 2). As summarized in Table 1, α7-nAChR expression was significantly highly positively correlated with the smoking status (*p* < 0.001), alcohol consumption (*p* = 0.03), liver cirrhosis (*p* = 0.001), tumor size (*p* < 0.001), tumor number (*p* = 0.01), vascular invasion (*p* < 0.001), tumor recurrence (*p* = 0.001), and pathological stage (*p* = 0.001), but not with age, gender, AFP, or the viral status. In addition to the above findings, our clinicopathological analyses showed that high α7-nAChR expressions were positively correlated with recurrent HCC (Spearman’s *r* = 0.60, *p* < 0.001) (Table 1). Representative IHC images of the α7-nAChR in non-cancerous and cancer tissues of different TNM stages are shown in Figure 2A. HCC specimens characterized as TMN stage III exhibited strong α7-nAChR immunopositivity; TMN stage II tissues showed moderate staining, stage I specimens showed mild staining, and corresponding normal liver (NL) tissues showed negative staining (Figure 2A). The scoring for α7nAChR staining and representative images of the different scoring intensities is shown in Supplementary Materials Figure S1. A statistical analysis of the immunopositivity of TMN stages revealed significant serial hierarchical differences among clinical stages (IV > III > II > I, *p* < 0.001: where for IV vs. I, *p* < 0.001; III vs. I, *p* < 0.05; and II vs. I, *p* < 0.05) (Figure 2B); furthermore, we observed significantly higher immunopositivity in late-stage HCC cases (*p* < 0.001). At the 5-year follow-up, high expression of the α7-nAChR was significantly correlated with reduced OS for stage I (50.2 vs. 33.8 months), stage II (36.7 vs. 19.8 months), and stage III (34.7 vs. 6.8 months) (*p* < 0.001), respectively (Figure 2C–E, Table 1). For the entire cohort (*n* = 179), the OS and disease-free survival (DFS) rates for low and high expressions were 50.1 and 13.8, and 46.3 and 11.2, respectively, and high α7-nAChR expression significantly decreased OS and DSF rates (*p* < 0.001, Figure 2F,G). In the univariate analysis of the smoking status, we found that alcohol consumption, liver cirrhosis, tumor size, tumor type, vascular invasion, and TNM stage were prognostic factors for OS and recurrence (Table 2). A multivariate analysis was used to determine whether the α7-nAChR was an independent factor for HCC. Significant factors in the univariate analysis were selected for the multivariate analysis. The α7-nAChR was shown to be an independent prognostic factor for OS (*p* < 0.001) and DFS (*p* < 0.001). HCC patients with high α7-nAChR expression were 2-fold more likely to experience recurrence (95% confidence interval (CI), 1.08–4.21; *p* = 0.001) than were patients with low α7-nAChR expression (Table 2).

### 3.4. α7-nAChR Promotes Cell Viability, Metastasis, and CSCs-Like Phenotypes in HCC

**Figure 3:**
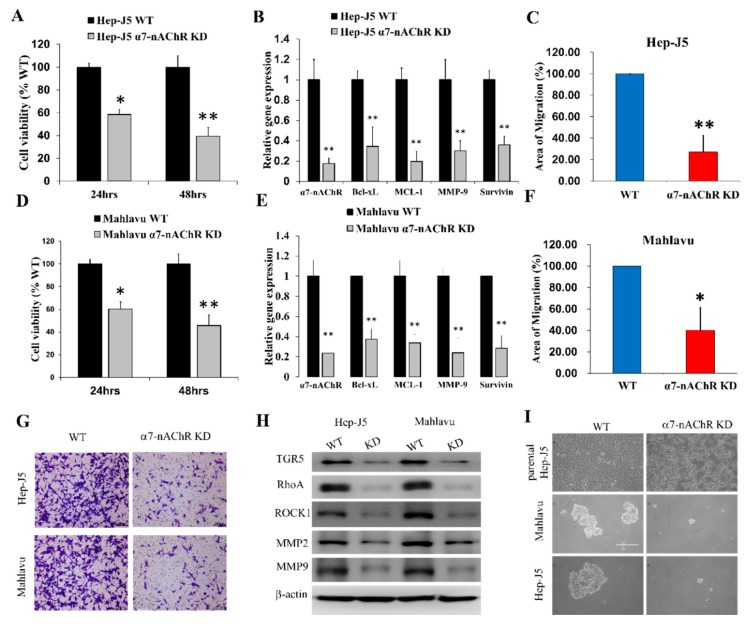
The α7-nicotinic acetylcholine receptor (α7-nAChR) promotes cell viability, metastasis, and cancer stem cell (CSCs)-like phenotypes in hepatocellular carcinoma (HCC). (**A**) α7-nAChR-knockdown (KD) inhibited the cell viability of Hep-J5 cells, as analyzed by an SRB assay after 24 and 48 h of incubation. * *p* < 0.05, ** *p* < 0.01, *p* < 0.001 versus the wild-type (WT) groups (*n* = 3). (**B**) Graphical representation of the inhibitory effect of α7-nAChR-KD on the mRNA expressions of α7-nAChR, Bcl-xL, MCL-1, MMP-9, and Survivin in Hep-J5 cells. (**C**) α7-nAChR-KD cells exhibited slower area closure than did Hep-J5 WT cells in the in vitro Scratch-wounding cell migration assay (*n* = 3) after 48 h. (**D**–**F**) similar results were obtained in Mahlavu cells. (**G**) Representative photo-image showing the effect of α7-nAChR-KD on the invasive potential of Hep-J5 and Mahlavu cells. (**H**) Differential expressions of TGR5, RhoA, ROCK1, MMP2, and MMP9 proteins in Hep-J5 and Mahlavu WT and α7-nAChR-KD cells. (**I**) α7-nAChR-KD Mahlavu and Hep-J5 cells exhibited a significantly reduced ability to form tumorspheres compared to WT cells. The assays were performed in triplicate, and data shown are representative of three independent assays. β-Actin served as a loading control.

To further investigate the biological functions of the α7-nAChR in HCC using scrambled control and α7-nAChR-KD Hep-J5 and Mahlavu cells, we demonstrated that loss of α7-nAChR expression significantly diminished HCC cell viability as expressed by 41% (*p* < 0.05) and 60% (*p* < 0.01) reductions in the population of viable Hep-J5 cells after 24 and 48 h, respectively (Figure 3A). Concurrent with the suppression of the α7-nAChR mRNA expression level, in Hep-J5 α7-nAChR-KD cells, we observed significant downregulation of Bcl-xL, MCL-1, Survivin, and MMP-9 mRNA expression levels (*p* < 0.01) (Figure 3B). The effect of the α7-nAChR on the migration of HCC cells was evaluated using an in vitro scratch-wound assay. Silencing the α7-nAChR remarkably suppressed the migratory activity of Hep-J5 cells (Figure 3C). The same results were also observed in Mahlavu cells; loss of α7-nAChR significantly decreased cell viability as expressed by 45% (*p* < 0.05) and 56% (*p* < 0.01) after 24 and 48 h, respectively (Figure 3D). Suppression of the α7-nAChR mRNA expression level significantly downregulated the expression of Bcl-xL, MCL-1, Survivin, and MMP-9 mRNA (*p* < 0.01) (Figure 3E). Migratory activity was also suppressed in Mahlavu cells (Figure 3F). To examine the effect of the α7-nAChR on the invasive ability of HCC cells, we performed invasion assays and found that knockdown of α7-nAChR significantly reduced the number of invading Hep-J5 and Mahlavu α7-nAChR-KD cells compared to scrambled control cells (Figure 3G). The observed suppression of migration and invasion in α7-nAChR-KD cells was associated with concurrent downregulation of TGR5, RhoA, ROCK1, MMP2, and MMP9 protein expression levels (Figure 3H). Furthermore, since liver CSCs were implicated in the formation, invasion, and metastasis of HCC cells [21], we evaluated the effect of inhibiting α7-nAChR expression on the CSCs-like traits of HCC cells. Results of our tumorsphere formation assay demonstrated that silencing the α7-nAChR significantly suppressed the ability of Hep-J5 and Mahlavu cells to form tumorspheres, which are in vitro CSCs models (Figure 3I). These results indicated that the α7-nAChR modulates the viability, migratory, invasive, and CSCs-like phenotypes of HCC cells in vitro.

### 3.5. The α7-nAChR’s Oncogenic Role Is Mediated in Part by JAK2 Activation

**Figure 4:**
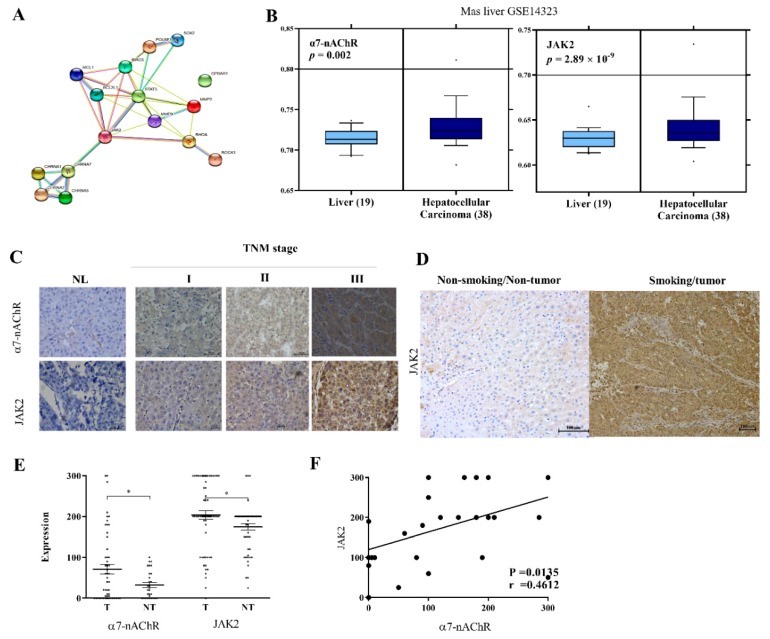
The oncogenic role of the α7-nicotinic acetylcholine receptor (α7-nAChR) is mediated in part by JAK2 activation. (**A**) Visualization of the molecular connectivity network among the α7-nAChR, JAK2, STAT3, SOX2, OCT4A (POU5F1), Survivin (BIRC5), Bcl-xL (BCL2L1), MCL-1, MMP2, MMP9, RhoA, and ROCK1 using STRING version 10.5. (**B**) Differential expressions of the α7-nAChR and JAK2 in hepatocellular carcinoma (HCC) specimens compared to non-tumor liver specimens in the GSE14323 dataset. (**C**) Representative images of α7-nAChR and JAK2 expression levels in HCC tissues of different TMN stages showing mild expression in stage I, moderate expression in stage II, and high expression in stage III, compared to no expression in non-tumor liver (NL) tissues. The original magnification is indicated; bar = 100 μm. (**D**) Photo-images showing the differential expression of JAK2 in HCC specimens from a current smoker compared to non-tumor liver specimens from a non-smoker. (**E**) Dot and whisker plots showing that the α7-nAChR and JAK2 are upregulated in tumor compared to non-tumor liver tissues. * *p* < 0.05. (**F**) Graph showing the positive correlation between the α7-nAChR and JAK2 in patients with HCC.

We explored the molecular mechanism underlying the oncogenic activities of the α7-nAChR as demonstrated above. A molecular network analysis using STRING, version 10.5 (https://string-db.org/cgi/network.pl?taskId=EZPtaWlDl5wA) revealed that the α7-nAChR interacts with JAK2, thereby activating downstream effector genes of the latter, including STAT3, SOX2, OCT4A (POU5F1), Survivin (BIRC5), Bcl-xL (BCL2L1), MCL-1, MMP2, MMP9, RhoA, and ROCK1. While no physical bond was seen among the α7-nAChR (CHRNA7), JAK2, and TGR5 (GPBAR1), an association that was in part suggestive of a joint contribution to a shared function was noted (Figure 4A). Similarly, we evaluated a microarray dataset of human liver cancer GSE14323 for aberrantly expressed genes. We observed a positive correlation between expression profiles of α7-nAChR and JAK2 genes. The α7-nAChR was significantly overexpressed in HCC specimens (*n* = 38) compared to non-tumor liver specimens (*n* = 19) (~2-fold increase, *p* = 0.002); this was similar to that of JAK2 (~2-fold increase, *p* = 2.89 × 10^−9^) (Figure 4B). These results were corroborated by IHC staining, which showed positive correlations of concurrently increased α7-nAChR and JAK2 immunopositivity with an increased TNM stage (Figure 4C, Supplementary Materials Figures S1 and S2). As with the α7-nAChR, we demonstrated JAK2 immunopositivity in patients with HCC who were also current smokers, compared to very mild JAK2 immunopositivity in persons without HCC and who were non-smokers (Figure 4D). Statistical analyses showed a significantly positive correlation between α7-nAChR and JAK2 protein expressions in the 179 HCC patients (*p* = 0.01) (Figure 4E,F).

### 3.6. The α7-nAChR Promotes the Recurrence of Human HCC by Modulating the TGR5/JAK2/STAT3 Signaling Axis

**Figure 5:**
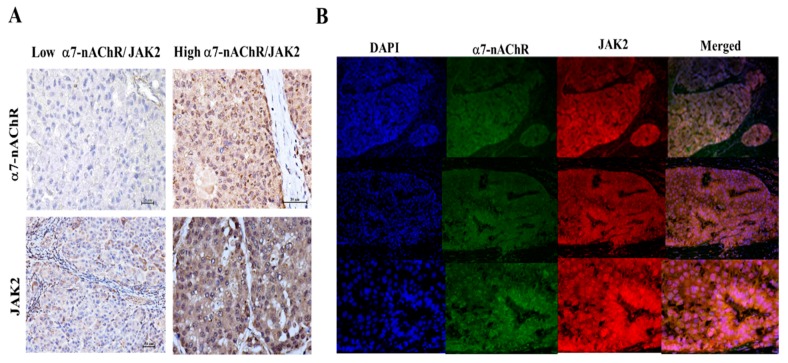

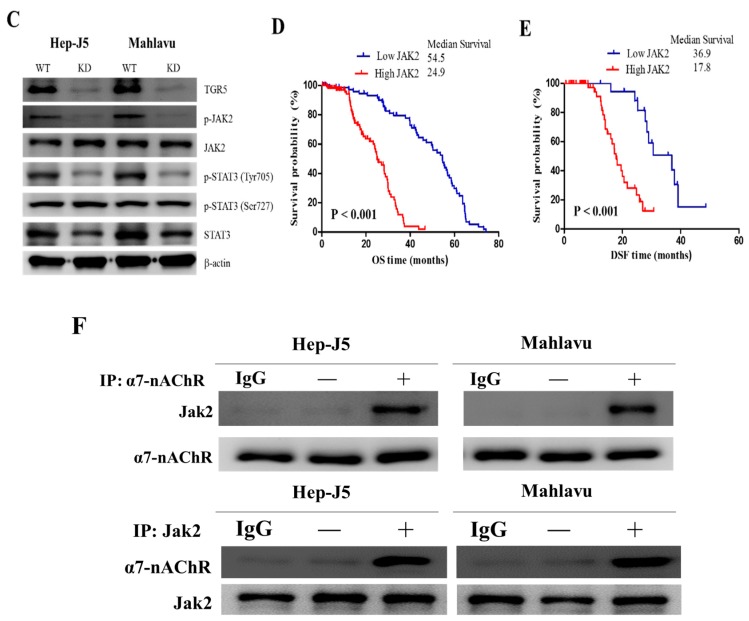
The α7-nicotinic acetylcholine receptor (α7-nAChR) promotes metastasis and recurrence of human hepatocellular carcinoma (HCC) by modulating the JAK2/STAT3 signaling axis. (**A**) Representative IHC photo-images showing the correlation of α7-nAChR expression levels on the immunoreactivity of TGR5 and JAK2 in HCC tissues. The original magnification is indicated; bar = 100 μm. (**B**) Nuclear co-localization of the α7-nAChR and TGR5 in HCC samples as indicated by IFC staining. DAPI served as a nuclear marker. (**C**) Western blot images showing the effect of silencing of the α7-nAChR on expressions of TGR5, p-JAK2, JAK2, p-STAT3 (Tyr705), p-STAT3 (Ser727), and STAT3. β-Actin served as a loading control. (**D**) Overall survival of JAK2 expressions. (**E**) Comparison of disease-free survival with low and high levels of JAK2 expression. (**F**) α7-nAChR/JAK2 complex formation was detected by immunoprecipitation (IP). Hep-J2 and Mahlavu cells lysate were incubated with α7-nAChR and JAK2 antibody, respectively. IgG was used as a negative control.

In addition to the above findings, our clinicopathological analyses showed that moderate-to-strong or high α7-nAChR and JAK2 expressions were positively correlated with recurrent HCC (*p* < 0.001). We also demonstrated that HCC tissues with low α7-nAChR expression were TGR5 immunonegative, unlike HCC tissues with high α7-nAChR expression, which were highly TGR5 immunopositive. This immunoreactivity profile was similar for JAK2 (Figure 5A). Furthermore, we showed that the α7-nAChR and JAK2 were highly expressed in HCC tissues and were co-localized in the cytomembrane (Figure 5B). Results of our Western blot analysis also demonstrated that compared to WT Hep-J5 and Mahlavu cells, expression levels of TGR5, p-JAK2, JAK2, p-STAT3 (Tyr705), and p-STAT3 (Ser727) proteins were significantly downregulated in α7-nAChR-KD cells (Figure 5C). Effects of high expressions of α7-nAChR and JAK2 on the OS and DFS of patients with HCC (Figure 5D,E) were significantly lower. We cultured and lysed the Hep-J5 and Mahlavu cells, immunoprecipitating α7-nAChR via antibody, and IgG as control. Immunoprecipitated proteins were separated by gel electrophoresis, transferred to PVDF membrance, and immunoblotted with anti-JAK2 antibody (Figure 5F). The resulted indicated α7-nAChR formed a complex with JAK2. A similar result was also obtained by using JAK2 antibody to immunoprecipitate the Janus kinase 2 protein and was recognized by the anti-α7-nAChR antibody in western blot (Figure 5F).

## 4. Discussion

Despite advances in cancer diagnostic and anticancer therapeutic strategies, HCC remains a leading cause of cancer-related death in patients, in large part due to HCC metastasis, disease recurrence, and the lack of a definitive cure for metastatic and recurrent HCC [31]. This present study determined the clinicopathological significance of the α7-nAChR as a novel prognostic marker for patients with HCC, with a particular focus on probable associations with CSCs, macrometastasis, and survival. Beyond neuronal cells, there is convergent evidence that exposure to smoking or nicotine increases α7-nAChR levels in non-neuronal cells such as normal lung epithelial cells, keratinocytes, monocytes, pancreatic ductal adenocarcinoma (PDAC) cells, and human squamous cell lung cancer cells [32,33].

In the present study, we showed that nicotine exposure induces elevated α7-nAChR levels in human HCC. We demonstrated that the α7-nAChR is nicotine-dependent and deferentially expressed between HCC and non-tumor liver tissues (Figure 1, Table 1). This is consistent with recent documentation of the involvement of continued nicotine exposure in α7-nAChR upregulation, and the role of the α7-nAChR in promoting lung cancer proliferation and facilitation of human squamous cell lung cancer tumor growth and progression [34]. We further showed that α7-nAChR expression is an independent prognosticator of patients with HCC. Our data revealed that α7-nAChR overexpression in HCC samples was significantly correlated with the TMN stage, a large tumor size, tumor number, vascular invasion, and shorter OS and DFS (Figure 2, Table 2, Supplementary Materials Figures S1 and S2). This is in concordance with contemporary knowledge that exposure to nicotine promotes cancer cell proliferation, angiogenesis, and the epithelial–mesenchymal transition (EMT), culminating in enhancements of tumor growth and metastasis [35]. In fact, it was recently shown that nicotine can induce invasion and migration in different cancer types including breast, pancreatic, lung, and gastric cancers through α7-nAChR-mediated oncogenic signaling [36].

Furthermore, the nicotine-induced α7-nAChR elicits alterations in gene expressions that are consistent with evasion of cell death, cancer cell survival, and metastasis, namely Bcl-xL, MCL-1, and Survivin, which belong to the BCL2 family of regulators of apoptosis that enhance cell survival via inhibiting apoptosis [37]; ROCK1 and RhoA, which are key regulators of cell polarity, cell adhesion, and cell motility [38]; as well as MMP2 and MMP9 (Figure 3). Considering that recurrence is a major cause of HCC-related deaths and that the α7-nAChR is a modulator of recurrence, we posit that based on our findings, the α7-nAChR, by interacting with ROCK1 and RhoA, facilitates the loss of HCC cell–cell or cell–membrane adhesion, thereby increasing their motility and invasiveness via activation of MMP2 and MMP9 activities, giving rise to HCC vascular penetration and survival in the circulation, subsequently enhancing the migration of HCC cells into new tissues, and forming distant tumor colonization.

Our survival analyses revealed that α7-nAChR expression was significantly associated with poor clinical outcomes. Our clinical findings do suggest that inhibiting α7-nAChR signaling exhibited an inhibitory role in cancer progression. Thus, therapeutic or molecular inhibition of the α7-nAChR could prohibit oncogenic and metastatic processes, such as evasion of apoptosis, the EMT, migration, and invasion, as demonstrated in our study where the α7-nAChR promoted cell viability, metastasis, and CSCs-like phenotypes in HCC (Figure 3). This has clinical significance because, in spite of the remarkable progress made in HCC diagnostics and therapeutic strategies, disease recurrence after surgery remains a therapeutic challenge [39,40], and this is not unconnected with the presence of HCC-CSCs. Thus, it is necessary to identify CSCs markers like the α7-nAChR in the context of the present study for screening and early identification of patients with HCC, who are at high risk of disease relapse and likely to benefit from postoperative adjuvant therapy.

We used a molecular network analysis and identified differentially expressed genes affected by the α7-nAChR (Figure 4). Interestingly, the α7-nAChR significantly interacted with JAK2, and thereby activates the downstream effector gene, STAT3. Furthermore, it is increasingly evident that the JAK2/STAT3 signaling pathway plays a critical role in tumor growth, proliferation, and metastasis of several malignancies, including lung cancer [28]. The JAK2-modulated transcription factor, STAT3, involved in immune responses, inflammatory processes, and cancer initiation, is activated by several cytokines, growth factors, and oncogenes. We thus investigated the hitherto unexplored probable oncogenic role of the α7-nAChR and its modulation of JAK2/STAT3 signaling in HCC progression and prognosis.

We also demonstrated that α7-nAChR’s oncogenic role is mediated in part by JAK2 activation and that the α7-nAChR promotes recurrence of human HCC by forming α7-nAChR/JAK2 complex and modulating the downstream TGR5/JAK2/STAT3 signaling axis (Figure 5). These findings were corroborated by a previous report that the α7-nAChR/JAK2 complex transduces signals to the Akt/PKB axis, resulting in neuroprotection. Meanwhile, Liu et al.’s. recent work showed that the membrane bile acid receptor, TGR5, not only induces but also drives the growth and migration of non-small cell lung cancer cells by activating JAK2/STAT3 signaling [41]. Besides, nicotine/cigarette smoke promotes metastasis of pancreatic cancer through α7-nAChR mediated Mucin-4 (MUC4) upregulation. Chronic exposure to nicotine or cigarette smoke leads to increased expression of MUC4 in malignant cancer through activation of the α7-nAChR/JAK2/STAT3 and the MEK/ERK1/ERK2 signaling cascade [42]. Furthermore, tobacco smoking induces chronic inflammation to trigger the development of malignant cancer [43]. This is consistent with mounting evidence that the α7-nAChR plays a critical role in malignancies, to which our present findings add further insights. This study is limited by its relatively small cohort size and its retrospective nature; therefore, a prospective study with a large cohort size is needed in the future to further validate and establish the generalizability of our findings.

## 5. Conclusions

We revealed a potential HCC-relevant oncogene, the α7-nAChR, and demonstrated that nicotine-induced upregulation of the α7-nAChR promotes metastasis and recurrence of human HCC by modulating the JAK2/STAT3 signaling axis, while independently prognosticating clinical outcomes of patients with HCC. Thus, these findings suggest a potential role of the α7-nAChR as a novel molecular target in the treatment of HCC.

## Figures and Tables

**Table 1:** Clinicopathological correlations of α7-nAChR expressions in hepatocellular carcinoma (HCC) patients determined by immunohistochemistry (IHC).

Parameters	Expression, *n* (%)			Spearman’s Correlation		5-Year SR ^c^		
	Low	High	*p* ^a^	*r*	*p*^b^ value	Low	High	*P* ^d^
	69 (38.5)	110 (61.5)						
**Age (years)**								
<60	51 (73.9)	71 (64.5)	0.13	−0.10	0.39	55.6	9.1	0.28
≥60	18 (26.1)	39 (35.5)				39.8	14.2	
**Gender**								
Male	53 (76.8)	90 (81.8)	0.27	−0.16	0.04	50.1	9.5	0.03
Female	16 (23.2)	20 (18.2)				28.6	3.7	
**Smoking Status**								
smokers	49 (71.0)	41 (37.6)	<0.001	0.71	0.004	55.6	19.5	<0.001
Ex-smokers	12 (17.4)	24 (21.8)				28.4	6.4	
Current-smokers	8 (11.6)	45 (40.9)				27.0	5.9	
**Smoking pack-years**								
Non smokers	50 (72.5)	40 (36.4)	<0.001	0.67	<0.001	50.3	8.6	0.001
>0 and <20	10 (14.5)	35 (31.8)				29.5	6.8	
≥20	9 (13.0)	35 (31.8)				29.6	5.8	
**Alcohol consumption**								
No	46 (66.7)	46 (41.8)	<0.001	0.56	0.03	55.6	14.8	0.02
Low–Moderate	17 (24.6)	33 (30.0)				39.4	6.8	
Moderate–High	6 (8.7)	31 (28.2)				34.5	6.1	
**Alpha-fetoprotein (ng/mL)**								
<20	43 (62.3)	67 (60.9)	0.44	0.09	0.23	29.7	20.5	0.29
≥20	26 (37.7)	43 (39.1)				28.4	16.5	
**Viral status**								
Negative	27 (39.1)	39 (35.5)	0.87	0.52	0.49	54.7	14.4	0.76
HBV	30 (43.5)	54 (49.1)				42.3	25.1	
HCV	10 (14.5)	15 (13.6)				42.4	17.8	
HBV & HCV	2 (2.9)	2 (1.8)				28.9	29.1	
**Liver cirrhosis**								
Negative	63 (91.3)	36 (32.7)	<0.001	0.6	0.001	47.8	14.2	
Positive	6 (8.7)	74 (67.3)				47.5	28.1	
**Tumor size**								
<5 cm	54 (78.3)	10 (9.1)	<0.001	0.46	<0.001	46.6	21.1	0.002
≥5 cm	15 (21.7)	100 (90.9)				47.2	18.1	
**Tumor number**								
Solitary	58 (84.1)	52 (47.3)	<0.001	0.53	0.01	46.7	31.3	0.05
Multiple	11(15.9)	58 (52.7)				43.8	9.8	
**Vascular Invasion**								
0	50 (72.5)	22 (20.0)	0.001	0.6	<0.001	46.6	31.5	0.03
1	12 (17.4)	31 (28.2)				59.1	23.6	
2	5 (7.2)	42 (38.2)				41.9	11.7	
3	1 (1.4)	2 (1.8)				33.2	11.1	
4	1 (1.4)	13 (11.8)				35.3	10.8	
**Recurrent**								
No	50 (72.5)	54 (49.1)	0.002	0.60	0.001	42.8	13.3	<0.001
Yes	19 (27.5)	56 (50.9)				51.2	27.7	
**TNM stage**								
I	55 (79.9)	10 (9.1)	<0.001	0.69	<0.001	50.2	33.8	0.05
II	10 (14.5)	49 (44.5)				36.7	19.8	0.001
III	3 (4.3)	43 (39.1)				34.7	6.8	0.01
IV	1 (1.4)	8 (7.3)				3.5	2.3	0.02

^a^ Mann–Whitney U test (for 2 groups) or Kruskal–Wallis test (for >2 groups), ^b^ Spearman Correlation, ^c^ Five-year survival rate, ^d^ Log-rank test. HCV: hepatitis C, HBV: hepatitis B.

**Table 2:** Univariate and multivariate analyses of factors associated with survival and recurrence.

Parameters	Univariate	Overall Survival Multivariate		Univariate	Disease Free Survival Multivariate	
	*p*	HR (95% CI)	*p*	*p*	HR (95% CI)	*p*
Age (<60 vs. ≥60)	0.459			0.35		
Gender (male vs. female)	0.132			0.02		
Smoking status (yes vs. no)	<0.001	2.02 (1.71–3.37)	<0.001	0.01	1.31 (1.32–2.66)	0.025
Smoking pack—years (light vs. heavy)	0.001	2.19 (1.56–3.52)	0.02	0.001	1.5 (1.51–2.05)	0.023
Alcohol consumption (low vs. high)	0.009	1.02 (1.14–2.62)	0.01	0.001	1.6 (1.51–2.32)	0.002
AFP (<20 vs. ≥20 ng/mL)	0.92			0.65		
Viral status (yes vs. no)	0.13			0.34		
Liver cirrhosis (yes vs. no)	0.02	1.51 (1.22–1.74)	0.002	0.001	1.02 (1.14–2.62)	
Tumor size (<5 cm vs. ≥5 cm)	0.04	1.23 (1.14–2.15)	<0.001	0.001	1.8 (1.73–2.05)	0.028
Tumor type (solitary vs. multiple)	<0.001	1.71 (1.74–2.13)	0.05	0.001	1.3 (1.12–2.61)	<0.001
Vascular Invasions (yes vs. no)	0.008	1.59 (1.35–2.13)	0.002	0.01	2.46 (1.32–3.84)	0.008
TNM stage (I/II vs. III/IV)	0.01	1.01 (1.21–1.86)	<0.001	0.01	1.21 (1.81–2.01)	0.012
α7-nAChR (low vs. high)	<0.001	2.01 (1.76–3.42)	<0.001	0.001	2.62 (1.08–4.21)	0.001

AFP α-fetoprotein, Smoking pack—years; Light smoker (PY > 0 and < 20), Heavy smoker (PY ≥ 20), Alcohol consumption: low-moderate (1–50 g/day), medium-high drinker (>50 g/day).

## Supplementary Materials

The following are available online at https://www.mdpi.com/2077-0383/8/9/1391/s1, Figure S1: representative images of the different scoring intensities by using the quantitative and qualitative scoring systems. (**A**) Score 0: negative staining and negative positive staining cells. (**B**) Score 1: weak staining and <10% positive staining cells. (**C**) Score 2: moderate staining and 10-50 % positive staining cells. (**D**) Score 3: strong staining and >50 % positive staining cells, Figure S2: Representative foci stained α7-nAChR is highly expressed in tumor but not normal liver.
