# Supplementary material for: Smoking as an Independent Risk Factor for Hepatocellular Carcinoma Due to the α7-Nachr Modulating the JAK2/STAT3 Signaling Axis
Source: J Clin Med. 2019 Sep 5;8(9):1391. doi: 10.3390/jcm8091391 (PMC6780871; doi:10.3390/jcm8091391)
Supplement: Supplementary file 1 [file jcm-08-01391-s001.pdf]

SUPPLEMENTARY INFORMATION

Smoking as an independent risk factor for hepatocellular carcinoma due to the α7-nAChR modulating the JAK2/STAT3 signaling axis

Ching-Li Li[1,2], Yen-Kuang Lin[3], Hsin-An Chen[4,5], Chien-Yu Huang[4], Ming-Te Huang[1,4,5*], Yu-Jia Chang[1,6*]

[1]Graduate Institute of Clinical Medicine, College of Medicine, Taipei Medical University, Taipei, Taiwan, ROC

[2]Department of Surgery, Sijhih Cathay General Hospital, New Taipei City, Taiwan, ROC

[3]Biostatistics Center, Taipei Medical University, Taipei, Taiwan, ROC

[4]Department of Surgery, Taipei Medical University, Shuang Ho Hospital, New Taipei City, Taiwan, ROC

[5]Division of General Surgery, Department of Surgery, School of Medicine, College of Medicine, Taipei Medical University, Taiwan, ROC

[6]International PhD Program in Medicine, Taipei Medical University, Taipei, Taiwan, ROC

[*] **Corresponding author**

Ming-Te Huang. No. 291, Zhongzheng Rd., Zhonghe District, New Taipei City 235, Taiwan (R.O.C); Tel/Fax: 886-2-22490088 Ext 8123. E-mail: 15729@s.tmu.edu.tw

Yu-Jia Chang. 250 Wu-Xing Street, Taipei City 11031, Taiwan; Tel/Fax: +886-2-27361661 ext. 3027. E-mail: r5424012@tmu.edu.tw

Working title: Targeting the α7-nAChR inhibits liver cancer poor prognosis

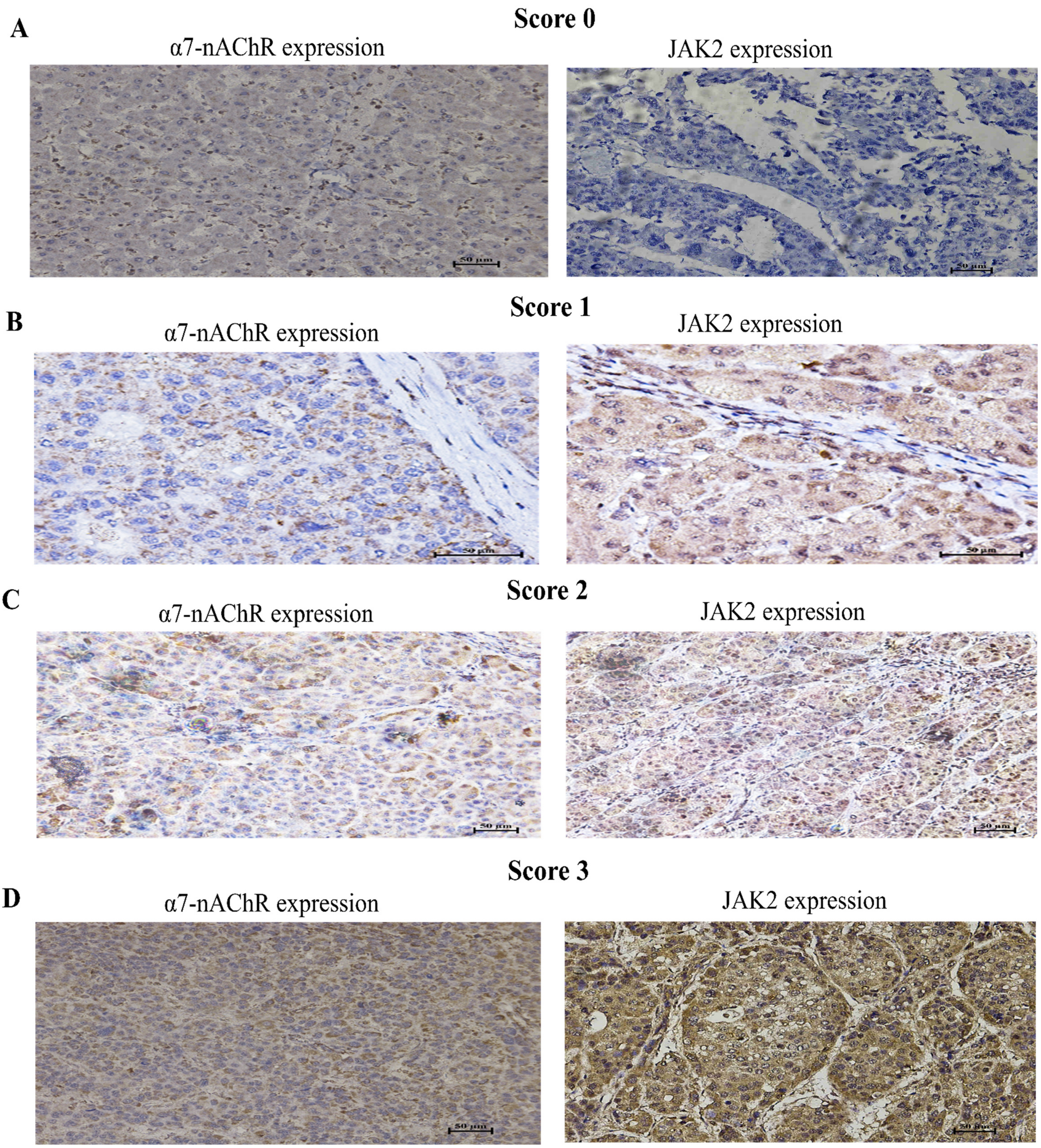

Supplementary Figure S1

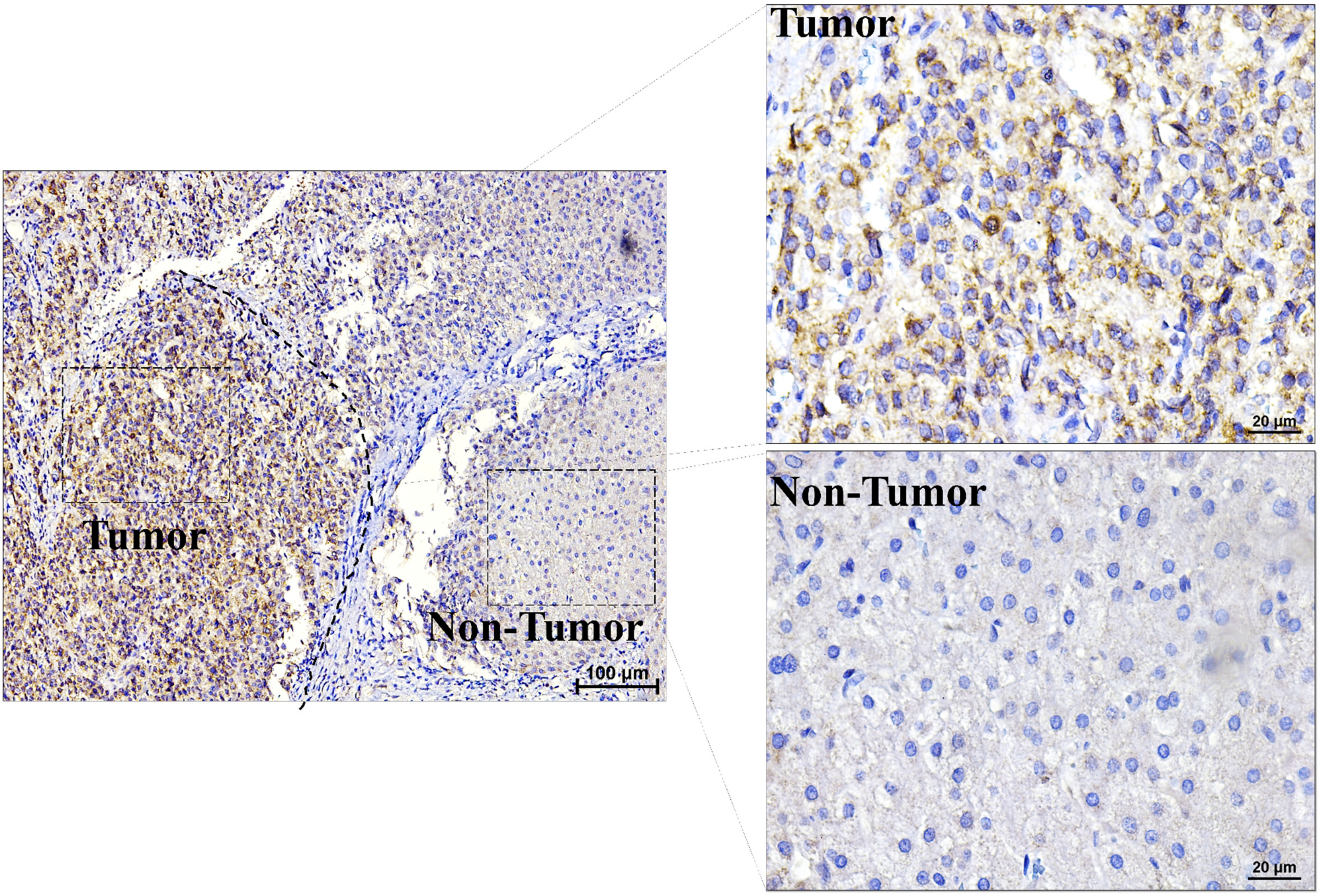

Supplementary Figure S2
